# Genome characterization of a multi-drug resistant *Escherichia coli* strain, L1PEag1, isolated from commercial cape gooseberry fruits (*Physalis peruviana* L.)

**DOI:** 10.3389/fmicb.2024.1392333

**Published:** 2024-07-22

**Authors:** Diana Molina, Julio C. Carrión–Olmedo, Pablo Jarrín–V, Gabriela N. Tenea

**Affiliations:** ^1^Biofood and Nutraceutics Research and Development Group, Faculty of Engineering in Agricultural and Environmental Sciences, Universidad Técnica del Norte, Ibarra, Ecuador; ^2^Laboratorio de Secuenciamiento de Ácidos Nucleicos, Dirección de Innovación, Instituto Nacional de Biodiversidad (INABIO), Quito, Ecuador

**Keywords:** cape gooseberry, antibiotic resistance, *Escherichia coli*, whole genome, food pathogens, beta-lactamase, *fim*H alleles

## Abstract

**Introduction:**

Foodborne infections, which are frequently linked to bacterial contamination, are a serious concern to public health on a global scale. Whether agricultural farming practices help spread genes linked to antibiotic resistance in bacteria associated with humans or animals is a controversial question.

**Methods:**

This study applied a long–read Oxford Nanopore MinION-based sequencing to obtain the complete genome sequence of a multi-drug resistant *Escherichia coli* strain (L1PEag1), isolated from commercial cape gooseberry fruits (*Physalis peruviana* L.) in Ecuador. Using different genome analysis tools, the serotype, Multi Locus Sequence Typing (MLST), virulence genes, and antimicrobial resistance (AMR) genes of the L1PEag1 isolate were determined. Additionally, *in vitro* assays were performed to demonstrate functional genes.

**Results:**

The complete genome sequence of the L1PEag1 isolate was assembled into a circular chromosome of 4825.722 Kbp and one plasmid of 3.561 Kbp. The L1PEag1 isolate belongs to the B2 phylogroup, sequence type ST1170, and O1:H4 serotype based on *in silico* genome analysis. The genome contains 4,473 genes, 88 tRNA, 8 5S rRNA, 7 16S rRNA, and 7 23S rRNA. The average GC content is 50.58%. The specific annotation consisted of 4,439 and 3,723 genes annotated with KEEG and COG respectively, 3 intact prophage regions, 23 genomic islands (GIs), and 4 insertion sequences (ISs) of the ISAs1 and IS630 families. The L1PEag1 isolate carries 25 virulence genes, and 4 perfect and 51 strict antibiotic resistant gene (ARG) regions based on VirulenceFinder and RGI annotation. Besides, the *in vitro* antibiotic profile indicated resistance to kanamycin (K30), azithromycin (AZM15), clindamycin (DA2), novobiocin (NV30), amikacin (AMK30), and other antibiotics. The L1PEag1 isolate was predicted as a human pathogen, matching 464 protein families (0.934 likelihood).

**Conclusion:**

Our work emphasizes the necessity of monitoring environmental antibiotic resistance, particularly in commercial settings to contribute to develop early mitigation techniques for dealing with resistance diffusion.

## Introduction

Foodborne illnesses, often attributed to bacterial contamination, represent a significant worldwide risk to public health. The role of agricultural farming systems in transferring antibiotic resistance genes to humans or animals is actively debated (Cheng et al., 2016; Tiedje et al., 2023). *Escherichia coli* is an important indicator organism in food and water contamination (Reid et al., 2020). A query to NCBI shows that whole genome sequences of *E. coli* from animals and associated meats are frequent, while sequences from food products, like fruits and vegetables, are rare. Food products may acquire drug-resistant *E. coli* from animal manure fertilizers, contaminated irrigation water, and other sources (Possas and Pérez-Rodríguez, 2023). Among the different strains of *E. coli*, certain pathogenic strains are known to cause gastrointestinal infections, ranging from mild to severe, and can lead to life-threatening complications (Thomas et al., 2024). Understanding their transmission, virulence factors, and mechanisms of pathogenesis is crucial for effective prevention and control strategies (Melo and Quintas, 2023).

Emergent antibiotic resistance among *Enterobacter* species is a source of significant concern, compromising the effectiveness of antimicrobial treatment options (Zurita et al., 2020). The inappropriate use of antibiotics in human and veterinary medicine, as well as in agriculture, has contributed to the selection and spread of resistant strains (Mann et al., 2021). Bacteria found in the human gut, such as *E. coli,* are frequently shown to be the root cause of drug-resistant infections (Manges and Johnson, 2012). The human gut microbiota is largely influenced by food, and this ecosystem is also a hotspot for horizontal gene transfer (HGT), where genetic components of antibiotic resistance may be traded between commensals and opportunistic infections (Coque et al., 2023). Even though almost all therapeutically significant antimicrobial medications are effective on *E. coli*, this pathogen has a strong probability of acquiring resistance genes, mainly via HGT (Paitan, 2018; Poirel et al., 2018; Arbab et al., 2022). The development of resistance mechanisms to extended-spectrum β-lactamases (ESBLs), which confer resistance to broad-spectrum cephalosporins, Metallo-β-lactamases enzymes (MBLs), which confer resistance to carbapenems, 16S rRNA methylases, which confer pan-resistance to aminoglycosides, and *mcr* genes, which confer resistance to polymyxins are among the most problematic mechanisms in *E. coli* (Poirel et al., 2018).

Among the many plant foods, fruits are a significant source of the vital elements needed for a healthy diet. Additionally, they offer bioactive phytochemicals, including flavonoids and phenolic compounds, which have been linked to several health advantages (Melo and Quintas, 2023). The microbiological safety of fruits has raised significant concerns in the food industry and public health agencies, and it is one of the most rapidly expanding sectors in the last years (Mostafidi et al., 2020). Due to the lack of preventive microbiological techniques to ensure the elimination or destruction of pathogens before consumption, such food products may expose consumers to risk of contracting foodborne infections (Pérez-Lavalle et al., 2020). Several reports have indicated the presence of pathogenic strains of *Salmonella enterica*, *E. coli*, and *Listeria monocytogenes* in minimally processed foods (Solomon et al., 2002; Strawn et al., 2011; Yoo et al., 2015; Pérez-Lavalle et al., 2020).

*Escherichia coli*-related outbreaks, associated with the consumption of fresh produce, have been increasing in frequency worldwide, as this microorganism is particularly plastic and resilient in its adaptations to survive on a wide variety of fruits, vegetables, environments, and sanitation methods (Luna-Guevara et al., 2019). Enteric pathogens such as *E. coli,* that are often involved in these outbreaks, have been understudied, emphasizing notable gaps in our understanding on the physiology and adaptations of human enteric pathogens, that are active or viable on agricultural produce (Lynch et al., 2008). A previous study recorded the presence of tetracycline resistant *E. coli* isolated from ready-to-eat, store-bought, mixed salad, arugula, and cilantro from two German cities (Reid et al., 2020). A recent study in Ecuador described the potential of irrigation water systems and agricultural products as a source of beta-lactam resistant *E. coli*, with 11% of the sampled vegetables positive for *E. coli* and 58% of the 165 *E. coli* laboratory cultures with the ESBL phenotype (Montero et al., 2021). Salazar-Llorente et al. (2021) and Cárdenas et al. (2024) surveyed microbial presence on hundreds of street market food samples from Ecuador, including fruits, their findings suggest poor sanitation and lack of clean water for food processing.

Ecuador has a high rate of antibiotic resistance in poultry and water systems (Molina et al., 2024), which requires the creation of regulations and guidelines for the use of antibiotics (Amancha et al., 2023). To better track the use of antibiotics and changing resistance patterns, the current surveillance system must be enhanced. The bacteriological safety of fresh fruits in Ecuadorian agricultural farms (Montero et al., 2021) and low-cost markets was recently assessed (Tenea et al., 2023); since this produce is usually eaten raw, there is a greater chance that bacteria will survive and spread to the human gut, than when cooked food is consumed (Melo and Quintas, 2023).

Cape gooseberry (*Physalis peruviana*), locally known as uvilla or uchuva, is a super nutritive and exquisite fruit that requires attention, as it is contaminated with *Staphylococcus* spp. and several *Enterobacteriaceae*, including *E. coli* (Tenea et al., 2023). Among a pool of antibiotic resistant isolates, one isolate annotated L1PEag1 was selected, and the whole genome sequenced. Through various *in silico* tools, the isolate was typed, serotyped, and its evolutionary relationship assessed. With the assistance of web server tools, we predicted the presence and characteristics of antibiotic-resistant genes, virulence factors, CRISPR sequences, pathogenicity factors, and *fim*H alleles. *In vitro* assays (antibiotic sensitivity, hemolysis, gelatinase activity) were applied to confirm antibiotic resistance and pathogenicity. Besides exploring, characterizing, annotating, and describing a new genome of *E. coli* native to Ecuador, our research contributes to current efforts on drawing attention on food contamination with pathogenic bacteria and to the search for solutions to combat cross-contamination, helping to promote public health and ensure safer food systems.

## Materials and methods

### DNA extraction and genome sequencing

The isolate L1PEag1 was isolated from fruit peel (exocarp) of cape gooseberry, purchased from the local market of Ibarra city, northern Ecuador, and following a standard procedure (Tenea et al., 2023). The purified isolate was grown overnight on Luria Bertani (LB) agar (Oxoid, UK) at 37°C. Genomic DNA was extracted using a high molecular weight DNA extraction kit (Wizard Genomic DNA purification kit), and following the manufacturer indications (Promega, USA). The DNA was quantified, using a NanoDrop™ 2000 Spectrophotometer (Thermo Fisher, USA). The library was prepared using a Ligation Sequencing kit V14 (SQK-LSK114) (Oxford Nanopore Technologies Ltd., UK). High molecular weight DNA was nick–repaired and end–prepared with the NEBNext® Companion Module for Oxford Nanopore Technologies® Ligation Sequencing (E7180S). The end–preparation protocol phosphorylated 5’ends and dA-tailed 3′ ends of gDNA fragments. A magnetic bead clean-up of enzymes was performed before ligation. Sequencing adapter ligation was performed with the NEBNext® Quick Ligation Module (E6056). After ligation, a second magnetic bead clean–up was performed before sequencing. The library was sequenced on a MinION Mk1C sequencer and a Flow Cell version R10.4.1 (FLO-MIN114, Oxford Nanopore Technologies Ltd., UK). Sequencing run took 48 h and generated 998.45 k reads at an estimated coverage of 400x.

### Genome assembly

Raw sequence data was high-accuracy basecalled into fastq data (HAC) under the dna_r10.4.1_e8.2_400bps_hac@v4.2.0 model with the Dorado 0.2.1 package (Hariharan et al., 2020). Quality control was performed with NanoPlot version 1.41.3 (Coster et al., 2018) and FastQC version 0.12.1^1^. To reduce errors in assembly, we applied filtering and trimming with NanoFilt 2.8.0 (Coster et al., 2018). Reads with less than a quality value of Q14 were filtered out for the assembly. To allow for plasmid assembly, short reads were not filtered out. Trimming was performed on the first 20 bps for each end of the reads to eliminate adapters. A subsequent round of quality control was performed after the filtering and trimming. *De novo* assembly was made according to the long–read assembly pipeline and the Trycycler tool by Wick et al. (2021), these consist in estimating a final consensus assembly from multiple independent assemblies as input. The reduction of error during the final assembly process, assisted by the Trycycler pipeline and its algorithms, relies on the fact that multiple independent subset assemblies provide alternative sources of evidence that compensate previous assembly biases. In brief: (1) By randomly subsampling the original read set, sequence data was subsampled into 12 independent read sets. To maximize the independence of assembly results, each subset was independently assembled with different assembling tools. The assemblers used for this step were (a) the repeat graph assembler FLYE 2.9.2-b1786 (Kolmogorov et al., 2019), (b) Minipolish (Wick and Holt, 2020), and (c) Raven (Vaser and Šikić, 2021). (2) The different assemblies were clustered based on their k-mer content. For the case of the L1PEag1 isolate, clusters recovered two replicons that consisted of the main circular genome and one accompanying plasmid. (3) The clustered sequences (contigs) were then reconciled by normalizing their directionality or orientation and aligned to each other to repair circularization issues such as missing bases or overlapping bases. (4) Each cluster of sequences was rotated to a common starting sequence, preparing them for multiple sequence alignment. (5) A global multiple sequence alignment was produced for each cluster of sequences. (6) A definitive consensus sequence for each cluster was obtained based on the minimum total Hamming distance to optional or variable regions in the consensus and the best total alignment score. (7) Finally, the consensus sequence was polished according to the Medaka v.1.1.1.3 algorithms^2^.

### Typing and evolutionary relationship

The genome FASTA sequence was uploaded to the Type (Strain) Genome Server (TYGS) to conduct a complementary genome-based taxonomic study and a phylogenetic relationship inference (Meier-Kolthoff and Göker, 2019). The closest strain genome type was determined by comparing the L1PEag1 genome to all accessible strain genomes in the TYGS database using the MASH algorithm (Meier-Kolthoff and Göker, 2019). Strains with the shortest MASH distance were selected automatically. Thus, the precise distance was calculated using the Genome BLAST distance phylogeny (GBDP) approach under the “coverage” algorithm and distance formula (Meier-Kolthoff et al., 2013). Phylogroups were determined using the *in silico* Clermont Phylotyper EzClermont^3^ (Waters et al., 2020). In addition, multi-locus sequence typing (MLST)^4^ (Larsen et al., 2012) was used for accurate subtyping, with the *E. coli* # 1 (Wirth et al., 2006), and *E. coli* # 2 (Jaureguy et al., 2008) schemes. Further, SerotypeFinder 2.0^5^ was used to identify the serotype (Joensen et al., 2015), using 95% sequence identity and 60% sequence coverage setup. To enrich the phylogenetic contrast, an additional genome search for similar *E. coli* sequences from Ecuador was made at NCBI’s databases and guided by previous published work (Montero et al., 2021; Rothstein et al., 2023). While genome sequences from earlier research have been made available at NCBI, these are either assembly-level contigs or raw sequences (SRA experiments in NCBI terminology), which do not meet the requirements for phylogenetic comparisons of the type carried out in this work on the whole assembled genome.

### Gene prediction and functional annotation

The L1PEag1 genome was annotated using the PROKKA tool (Seemann, 2014). Plasmid annotation was performed with pLannotate (McGuffie and Barrick, 2021). The predictions of CDS, rRNA, tRNA/tmRNA, signal leader peptide, and noncoding RNA were performed using Prodigal (Hyatt et al., 2010), RNAmmer^6^, Aragorn^7^, Signal IP^8^ and Infernal (Nawrocki and Eddy, 2013). Additionally, the predicted genes were annotated through the Global Catalog of Microorganisms genome annotation project pipeline^9^ and based ondatabases SwissProt^10^, MetaCyc^11^ (this is a database that contains pathways responsible for both primary and secondary metabolism) (Caspi et al., 2018), VFDB (a virulence factor database^12^) (Liu B. et al., 2019), PHI: The Pathogen-Host Interaction database^13^, KEGG (Kyoto Encyclopedia of Genes and Genomes), Orthology Database of Prokaryotes, COG (Clusters of Orthologous Groups of proteins), and pFam^14^ (Wu and Ma, 2019). A circular map was generated by importing the FASTA sequence into the PROKSEE server^15^ (Grant et al., 2023).

### Prediction of CRISPR sequences, prophage, and mobile elements

CRISPRFinder^16^ and PHAge Search Tool Enhanced Release (PHASTER)^17^ (Arndt et al., 2016) were used to detect CRISPR, Cas sequences, truncated Cas sequences, and prophage sequences. Only predicted prophages with intact completeness in PHASTER, as defined by Arndt et al. (2017), were considered for report. In addition, mobileOG-DB was used to annotate the mobile elements (Brown et al., 2022).

### Antibiotic-resistant genes, putative virulence genes, and pathogenicity prediction

The Comprehensive Antibiotic Resistance Database (CARD)^18^ (Jia et al., 2017) and the Resistance Gene Identifier tool (RGI) were used to detect the antibiotic resistance genes by importing the contig files in FASTA format to the database (Zankari et al., 2012). The ResFinder 4.3.3 server^19^ was used to identify acquired antimicrobial resistance genes (Bortolaia et al., 2020). The putative virulence factors were predicted using the VirulenceFinder 2.0 web server^20^ (Cosentino et al., 2013). The bacterial pathogenicity was predicted using the PathogenFinder web server^21^ (Cosentino et al., 2013). Whereas FimTyper 1.0^22^ was used to type de *fim*H alleles (Roer et al., 2017). The detection standard parameters were set at 90% sequence identity and 60% sequence coverage for VirulenceFinder and ResFinder.

### Identification of genomic islands (GIs) and insertion sequences (ISs)

GIs were predicted with the webserver IslandViewer 4, using *E. coli* SMS-3-5 as a reference strain (Bertelli et al., 2017). To search for ISs, the ISfinder tool (ISsaga V.2.0) was used (Siguier et al., 2006).

### *In vitro* assays

#### Hemolysis and gelatinase

For hemolysin production, plates containing 5% human blood agar was inoculated with the L1PEag1 isolate and incubated at 37°C for 24 h (Tabasi et al., 2015). The presence of partial or complete hemolysis was assessed on the plates. For gelatinase production, gelatin nutrient agar was used (Mittal et al., 2014). An overnight-old L1PEag1 inoculum was applied to the plate. Following observation of the organism’s growth, mercuric chloride solution was poured onto the plate. When a colony is flooded with mercuric chloride solution, the medium becomes opaque, and a clearing zone forms around it. This indicates that the colony is liquefying gelatin and is positive for gelatinase. As control, a human *E. coli* Ec1 (human enteropathogenic donated by Saint Vincente de Paul Clinical Hospital, Ibarra) and a human non-pathogenic *E. coli* ATCC25922 strains were also used.

#### Antibiotic profile

Antibiotic susceptibility was determined using the Muller-Hilton (MH) agar disk diffusion procedure, and according to the Clinical and Laboratory Standards Institute (CLSI) guidelines (EFSA Panel on Additives and Products or Substances used in Animal Feed et al., 2018). Briefly, 100 μL of inoculum (10^7^–10^8^ CFU/mL) was streaked onto MH plates. The commercial antibiotic disks (Merck, USA) were chosen as recommended by the National Plan for Surveillance and Control of Contaminants in Primary Production (NTE INEN 1529–2-2013)^23^. The disks were plated on MH agar plates, and incubated at 37°C for 48 h. The diameter of each clear zone was measured in millimeters by scanning the plates with a microplate reader (SCAN500, Interscience, Fr). *E. coli* ATCC25922 and *E. coli* UTNEc1 were used for quality control and comparison. The microbiological breakpoints reported by the FEEDAP standards were used to categorize *E. coli* as susceptible, intermediate, or resistant (EFSA Panel on Additives and Products or Substances used in Animal Feed et al., 2018).

#### Beta-lactamase resistance and virulence genes detection

Genomic DNA was extracted from the L1PEag1 isolate using the Wizard Genomic DNA purification Kit (# 1120 Promega, USA). The DNA concentration and purity were determined in a NanoDrop™ (Thermo Fisher Scientific, USA) at 230, 260, and 280 nm. The primers for *bla*_TEM_, *bla*_SHV_, *bla*_CTXM-2_, *bla*_CTXM-9_, *bla*_CTXM-8/25_, *bla*_NDM_, *bla*_KPC_, *bla*_VIM_, and *bla*_OXA-48/181_ genes were used at a concentration of 0.3 μM (Hernández-Alomía et al., 2023). The genetic determinants for virulence evaluated in this study were those that code for type 1 fimbriae (*fimH*), pili associated with pyelonephritis (*pap*), and S fimbriae (*sfa*) (Dadi et al., 2020). The sequences of these primers are listed in the Supplementary Table S1. The amplification was performed in reactions of 25 μL containing 2X GoTaq® Green Master Mix (#7132, Promega, USA) and the PCR reaction was performed in a Genemax Thermal Cycler (IQM, Olso, Norway). The amplification conditions for antibiotic resistance genes were as follows: denaturation step 2 min at 94°C, followed by 35 cycles of 1 min (denaturation) at 94°C, 1 min (annealing) at 50–60°C and 1.5 min (extension) at 72°C, and 1 cycle of 10 min (final step extension) at 72°C. For virulence genes, the amplification conditions were denaturation step for 4 min at 95°C, followed by 35 cycles of 40 s (denaturation) at 95°C, 45 s (annealing) at 53–65°C and 1 min (extension) at 72°C, and 1 cycle of 5 min (final step extension) at 72°C. The PCR products were separated by electrophoresis on 1% agarose gels in 1 x Tris-Borate EDTA (TBE, pH 8.0) buffer (Sigma-Aldrich Co., USA). Gels were stained in TBE buffer containing 0.5 μg/mL ethidium bromide. The results were registered as plus / minus for the presence of each amplicon.

## Results and discussion

### L1PEag1 isolate typing and phylogenetic relationship

**Figure 1:**
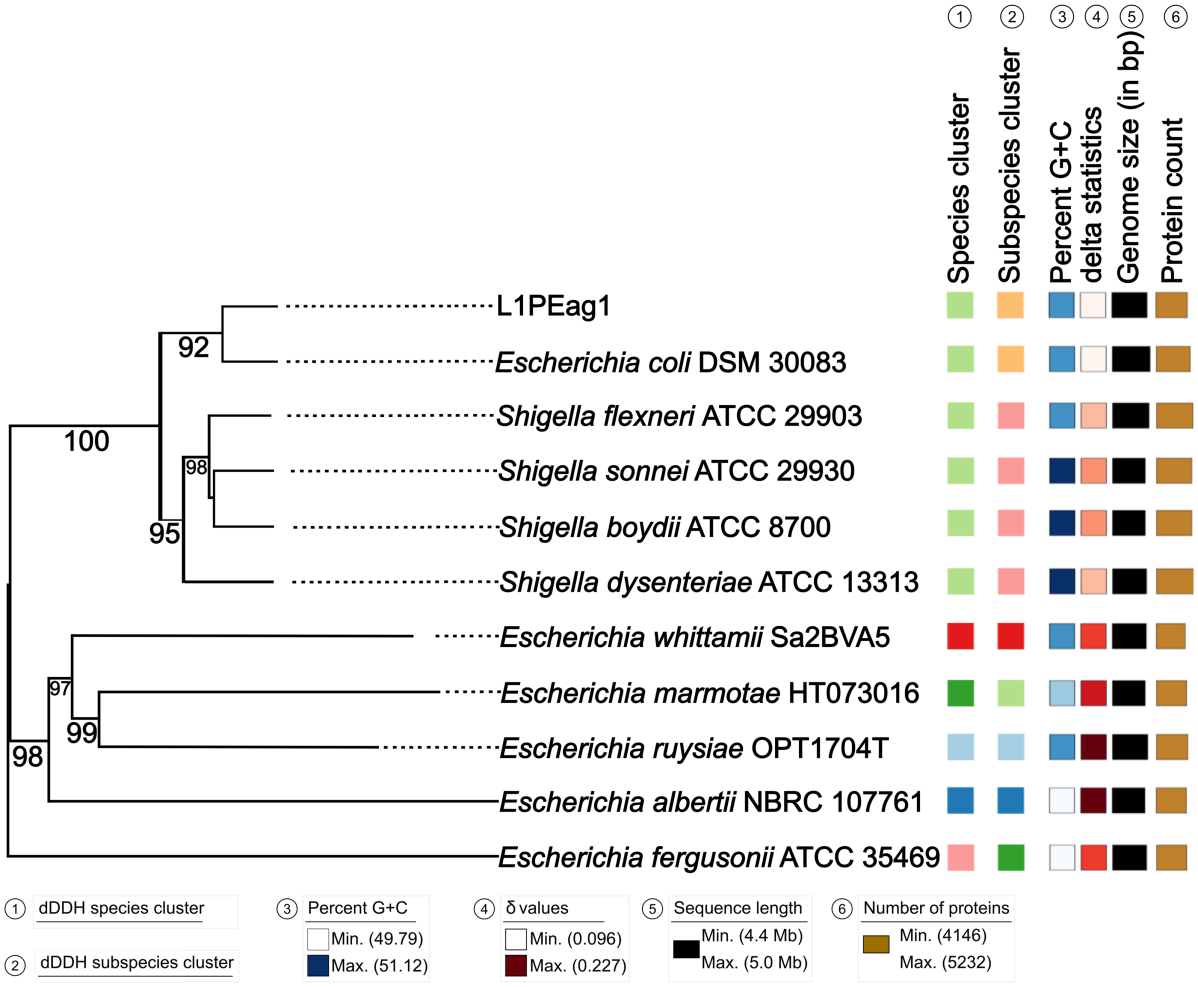
Phylogenetic tree based on TYGS results for the L1PEag1 whole-genome data set. Branch lengths are scaled in terms of GBDP (genome BLAST distance phylogeny method) distance; numbers below branches are GBDP pseudo-bootstrap support values from 100 replications. Leaf labels are annotated by affiliation to species clusters; subspecies clusters; genomic G + C content (min 49.79 – max 51.12); *δ* values (min 0.096 – max 0.227); overall genome sequence length (4,416,104 – 5,037,933); and number of proteins (min 4,146 – max 5,232).

An estimated genome size of 4825.72 Kbp (400X coverage) and a plasmid of 3.561 Kbp (400X coverage) were generated by the sequence analysis. Species matching resulted in a 100% hit on the entire contig for *Escherichia coli*. The results of the whole genome analysis placed the L1PEag1 strain on the same lineage as *E. coli* DSM30083 (Figure 1). Based on the BLASTN analysis, the plasmid pL1PEag1 showed 99.96% sequence identity with plasmid pECQ4552_IHU08 from *Klebsiella quasipneumoniae* subsp. *similipneumoniae,* strain IF3SW-P1 (CP092122.1); 99.89% identity with an unnamed plasmid from *Staphylococcus aureus* strain, Alexandria 2020–19 (CP113245.1); and 99.81% sequence identity with plasmid pECQ4552_IHU08 from *E. coli* strain Q4552 (CP077071.1).

### Phylogroup typing, MLST, and serotyping

Based on the Clermont Escherichia-typing method (Waters et al., 2020), the L1PEag1 isolate belongs to phylogroup B2 (*Tsp*E4 +, *chu +*, *yja*A +, and *arp*A –). Generally, B2 phylogroup strains are associated with Extraintestinal pathogenic *E. coli* (ExPEC) (Xia et al., 2022). A comparison of several *E. coli* strains prevalent in inflammatory bowel disease indicated that the B2 phylogroup strains share a specific metabolism and carry virulence genes enabling them to colonize intestinal mucosa (Fang et al., 2018). In *E. coli*, MLST requires assessment of 7 housekeeping genes (*adk, fum*C*, gyr*B*, icd, mdh, pur*A and, *rec*A) to assign sequence types (ST) genes (Wirth et al., 2006); thus, by using the *E. coli* #1 scheme, L1PEag1 was identified as the ST1170 sequence type. By using the *E. coli* #2 scheme (by the assessment of genes *din*B, *icd*A, *pab*B, *pol*B, *put*P, *trp*A, *trp*B, and *uid*A), no sequence type was discovered; nonetheless, the closest ST518 and ST966 were recommended (Supplementary Table S2). Several studies indicated the presence of specific STs in foods, whether of plant (ST2847, ST973) (Liu and Song, 2019; Song et al., 2020; Montero et al., 2021) or animal origin (ST1170) (Castellanos et al., 2017). *E. coli* isolates with phylogroup B2 and sequence type ST1170 were detected in wastewater from pig transport trucks (Savin et al., 2021) and from nosocomial and ambulatory infections in Germany (Pietsch et al., 2017). The ST966 type was identified in *Klebsiella pneumoniae* isolated from animal feed (Wu et al., 2022), suggesting environmental contamination. Cilantro-isolated *E. coli* samples, from a market in Germany, were classified as phylogroup B1 with multi-locus sequence types ST6186, ST165, ST58, and ST641 (Reid et al., 2020). Several *E. coli* strains, isolated from strawberries and banana obtained from agricultural farms in Ecuador, were classified into phylogroup B1, with the most frequent multi-locus sequence types being ST453, ST847, and ST6598 (Montero et al., 2021). Observed patterns of sequence types and phylogroups may be related with country- or producer-specific production methods, such as those pertaining to antimicrobial use, hygiene, and irrigation water systems (Molina et al., 2024). Based on the *fim*H typing, the L1PEag1 serotype ST1170 was classified as *fim*H20, with 100% sequence identity. Moreover, the subtyping results indicated the presence of the *fli*C gene, which matches the H-type H4 (100% identity with *E. coli* U1-41, AJ605764.1) (Beutin et al., 2005) and *wzy* genes (98.92% identity with the *E. coli* O1,H12 variant and 99.14% identity with the O1,H20 variant, KY115223.1). The presence of these variant alleles provided evidence for classifying the L1PEag1 isolate into the O1:H4 serotype. A single structural subunit (flagellin) encoded by the *fli*C gene defines the H antigen of *E. coli* (Reid et al., 1998), whereas the *wzy* gene encodes the O-antigen polymerase, which plays an important role in the synthesis of the lipopolysaccharide of bacteria (Zuo et al., 2019). The *E. coli* O1:H12 variant was detected in pig feces (Delannoy et al., 2017).

### Gene prediction and annotation

**Figure 2:**
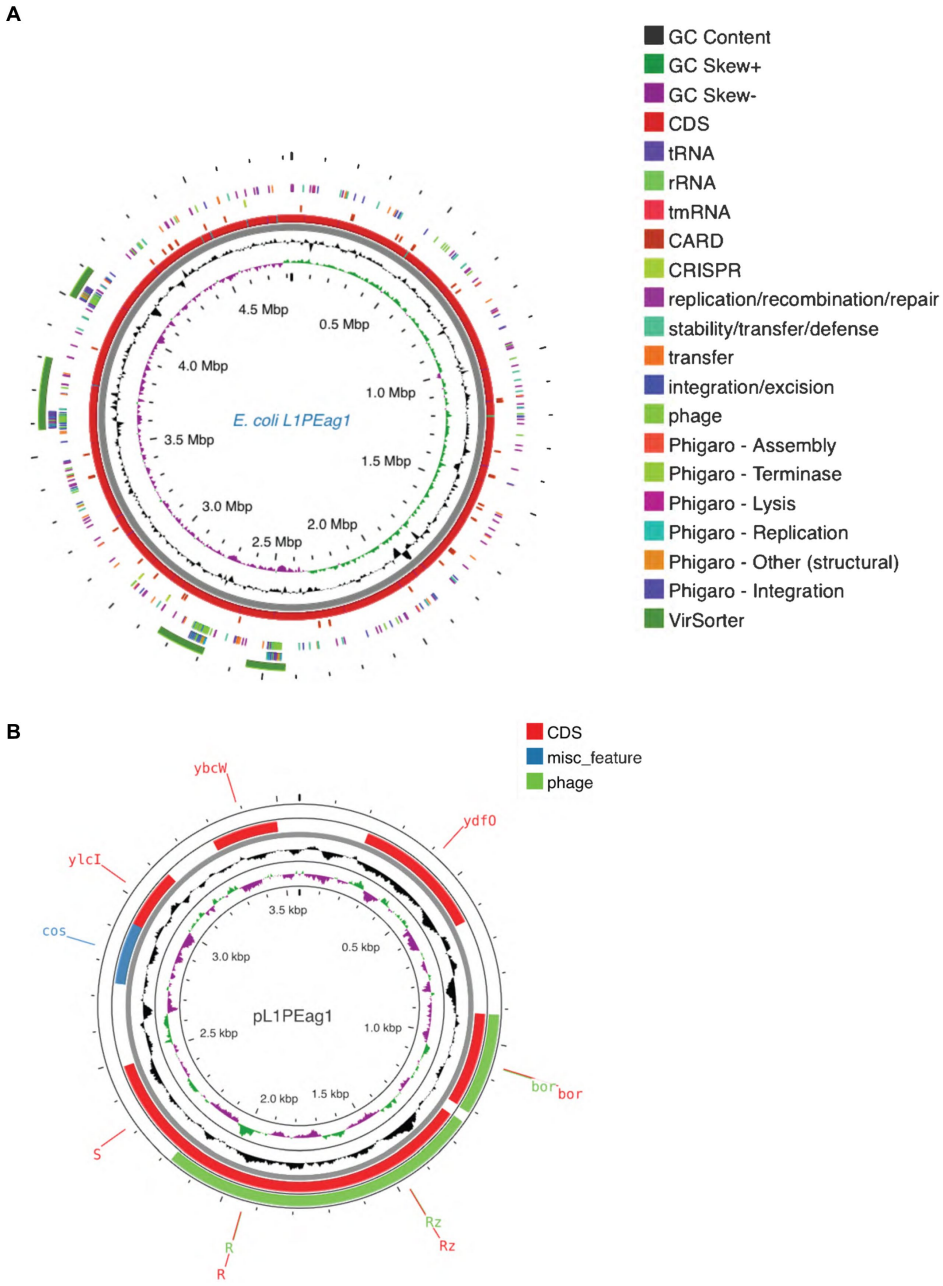
**(A)** Circular maps of the L1PEag1 genome predicted with the PROKSEE viewer (https://beta.proksee.ca/projects; Accessed on November 30th, 2023). The contents are arranged in feature rings. Starting with the outermost ring: Ring 1, prophage prediction with VirSorter annotation (mustard color); ring 2, mobile genetic elements (MGE) annotation with Alien Hunter predicting HGT (Horizontal Genetic Transfer) events (in red color); ring 3, prophage prediction with Phigaro annotation (genes involved in integration/replication/lysis/terminase are marked in different colors); ring 4, MGE annotation with Mobile OG DB marking the *hsdR* gene involved in stability/transfer/defense; rings 5–6 show the CDS (protein-coding genes) with Prokka annotation (blue color), tRNA, rRNA, and tmRNA are marked; ring 7 displays the GC content plot (black); ring 8 displays G/C skew information in the (+) strand (green color) and (−) strand (purple color). **(B)** Plasmid map as predicted by pLannotate (http://plannotate.barricklab.org).

The genome contains 4,473 genes, 88 tRNAs, and 22 rRNA (8 copies of 5S rRNA, 7 copies of 16S rRNA, and 7 copies of 23S rRNA). A physical genomic map is shown in Figure 2A. Prokka was used to predict the location, while BLAST was used to infer the function and identification of assembled sequences against nucleotide and protein sequence databases. Predicted genes in the previous step were aligned with several databases to obtain their corresponding annotations with the aligners (Table 1). The number of genes associated with COG (3,723 genes) and KEGG (4,439 genes) functional annotation categories are shown in Supplementary Figures S1A,B. Plasmid annotation (Figure 2B) consists of the following features: (1) R–region: an endolysin with trans glycosylase activity that degrades host peptidoglycans and participates with the holin and spanin proteins in the sequential events which lead to the programmed host cell lysis releasing the mature viral particles; (2) Rz: Component of the spanin complex that disrupts the host outer membrane and participates in cell lysis during virus exit (Berry et al., 2013; Rajaure et al., 2015); (3) *bor*-not known function, is expressed during lysogeny in *E. coli* (bacteriophage); (4) *ydf*O-putative protein (COG5562), 74.9% similarity with *E. coli* K-12 strain; (5) S- [Isoform Antiholin]: Counteracts the aggregation of the holin molecules and therefore pore formation; (6) cos: lambda cos site; allows packaging into phage lambda particles; (7) *ybc*W: Protein inferred from homology (*E. coli* O157:H7); (8) *ylc*I: unknown function, protein predicted in the reference genome of *E. coli* K12.

**Table 1:** Gene annotation summary.

**Num of genes (#)**	**CARD**	**MetaCyc**	**PHI**	**CAZy**	**VFDB**	**SwissProt**	**KEGG**	**COG**
4,473	135 (3.02%)	2032 (45.43%)	701 (15.67%)	178 (3.98%)	485 (10.84%)	3,834 (85.71%)	4,439 (99.24%)	3,723 (83.23%)

CARD, Comprehensive Antibiotic Resistance Database; MetaCyc, database that contains pathways involved in both primary and secondary metabolism; PHI, The Pathogen–Host Interaction database is a biological database that contains curated information on genes experimentally proven to affect the outcome of pathogen-host interactions (http://www.phi-base.org/searchFacet.htm?queryTerm); CAZy, Carbohydrate-active enzyme (http://www.cazy.org/); VFDB, virulence factor database (http://www.mgc.ac.cn/VFs/main.htm); SwissProt (https://www.uniprot.org/statistics/Swiss-Prot); KEGG: Kyoto Encyclopedia of Genes and Genomes; COG: Clusters of Orthologous Groups of proteins. The percentages in parentheses measure the proportion of coverage in the sequenced genome by the corresponding database.

### Prediction of CRISPR elements, prophage, virulence factors (VFs), genomic islands, insertion sequences, and pathogenicity

Two sequences with CRISPR were found within the L1PEag1 genome (beginning at position165680 and ending at 1656883 or at position 4,818,020 and ending at 4818143, respectively). Both encompass a short spacer sequence, spanning between degenerate repeats (consensus DRs) with 95.65 and 96.87% conserved repeats respectively, and 100% spacer conservation. No Cas elements were detected. There are seven identified prophage regions; of which three are complete, three are incomplete, and one is questionable. The Enterobacteria phages mEp460, SfV, and cdtI were the most frequently occurring intact phages (Table 2). Previous studies indicated the presence of Enterobacteria phage mEp460 in three *E. coli* strains isolated from seawater and marine sediment, indicating its high adaptability to different environments (Lai et al., 2017). Phage SfV is a temperate serotype-converting phage of *Shigella flexneri* and encodes the factors involved in type V O-antigen modification (Allison et al., 2002). *Shigella* phage SfIV and Enterobacteria phage cdtI (cytolethal distending toxins) were detected in the human gut microbiome and were associated with diarrhea (Federici et al., 2023). Additionally, a total of 23 GIs of 238,133 bp were predicted with IslandViewer using as a reference the multi-drug resistant *E. coli* SMS-3-5 genome (Fricke et al., 2008). A total of 229 hypothetical proteins, virulence genes, and antibiotic resistance genes were annotated in the GIs. Due to their frequent rearrangements, excisions, transfers, and further acquisition of additional DNA, these genomic regions play a significant role in the rapid evolution, diversification, and adaptation of *E. coli*. Most of them encode several proteins involved in the defense mechanism, virulence factors, iron-acquisition systems, and toxins (Supplementary Table S3). Our study included both *in vitro* and *in silico* approaches to analyze the pathogenicity of L1PEag1. The virulence factors detected *in silico* in the L1PEag1 genome are shown in Table 3. Among 25 genes from the virulence database, 8 genes showed perfect identity match (ID % = 100 match for the given gene, covering the entire length of the virulence genes in the database, 100% alignment), 12 were non-perfect matches (ID% < 100%, covering the entire length of the virulence genes in the database, 100 alignment), and 5 were non-perfect matches (ID < 85%, input sequence length is shorter than the virulence gene length, 60% alignment). It has been shown that virulence-associated regions are specific to carriage patterns, generally corresponding with specific *E. coli* STs. Out of 120 selected *E. coli* strains, 96% showed the presence of fimbrial adhesin gene *fim*H, whereas 63% contained glutamate decarboxylase gene *gad* (Reid et al., 2020). In our study, both virulence factors, *fim*H and *gad*, were detected with 100% identity. *fim*H is a type 1 fimbriae detected in more than 80% of *E. coli* strains and plays a significant role in the virulence of *E. coli* extraintestinal enteropathogenic strains (Poole et al., 2017). The enzyme GAD (glutamate decarboxylase) has been reported to be limited to pathogenic *E. coli*, being a virulence marker for contaminated food and water (Grant et al., 2001). In addition, the *yfc*V gene encodes a fimbrinal protein, the *Omp*T gene encodes an outer membrane protease (protein protease 7), the *fyu*A encodes a siderophore receptor, and *sit*A encodes an iron transport protein. In the L1PEag1 genome, all three genes, *Omp*T, *fyu*A, and *fim*H, were found with 100% match identity, within the island picks and were previously detected in urinary infections (Spurbeck et al., 2012; He et al., 2015). Although not found with a 100% match, the capsular polysaccharide gene *kps*M, serine protease autotransporter *vat,* and increased serum survival *iss*, which has been previously associated with ExPEC infection (Ravan and Amandadi, 2015), were detected in the target L1PEag1 genome. In addition, the virulence *fim*A, *fim*C, *fim*D, *fim*H, *fim*G, *foc*C, *kps*M, and *pap*C genes were annotated with Prokka. Complementary PCR analysis confirmed that L1PEag1 harbored a gene that encodes for type 1 fimbriae (*fimH*) and *pap* (pili associated with pyelonephritis) but not for S fimbriae (*sfa*) (Supplementary Table S1). This virulence gene profile was previously detected in *E. coli* isolated from urine culture of a patient with urine infection, and they belonged to phylogroup B2 (Rahdar et al., 2015).

**Table 2:** Prophage prediction with intact completeness, from genome analysis of the L1PEag1 isolate.

**Region**	**Region length**	**Completeness**	**Score #**	**Total Proteins**	**Region Position**	**Most Common Phage**	**GC %**
1	49.4Kb	Intact	150	65	595,182–644,618	Enterobacteria phage mEp460 (36)	49.21%
2	42.3Kb	Intact	150	59	1,017,491–1,059,827 0	Enterobacteria phage SfV (40)	50.18%
6	52.8Kb	Intact	150	56	2,158,903–2,211,727 0	Enterobacteria phage cdtI (10)	46.97%

All prophage predictions include intact completeness with a score of 150 as estimated by the PHASTER platform. Region: the number assigned to the region; region length: the length of the sequence in that region; completeness: a prediction of whether the region contains an intact or incomplete prophage based on the criteria: intact (score > 90%), questionable (score 70–90%), incomplete (score < 70%); score: the score of the region based on the mentioned criteria; # total proteins: the number of ORFs present in the region; Region position: the start and end positions of the region on the bacterial chromosome; Most common phage: the phages(s) with the highest number of proteins most similar to those in the region; GC%: the percentage of GC nucleotides in the region.

**Table 3:** *In silico* characteristics of virulence factors detected in the L1PEag1 genome.

**Virulence factor**	**% Identity**	**Query/HSP Length**	**Position in contig**	**Protein function**	**Accession number**	**Pathotype**
AslA	99.52	1,656/1,656	4751515.4753170	ND	CP022686	ExPEC
cdt-IVB	99.88	822/822	2206618.2207439	ND	CP000468	ExPEC
chuA	99.95	1983/1983	4409673.4411655	Outer membrane hemin receptor	UEKQ01000026	ExPEC
csgA	91.94	459/456	1811195.1811653	Curlin major subunit CsgA	CP069646	ExPEC
fdeC	91.82	4,217/4,254	1097195.1101408	Intimin-like adhesin FdeC	AP010953	ExPEC
fimH	100	489/489	549939.550427	Type 1 fimbriae	NA	ExPEC
fyuA	100	2022/2022	2787621.2789642	Siderophore receptor	UEKQ01000004	ExPEC
Gad	99.79	1,401/1,401	4428526.4429926	Glutamate decarboxylase	AP009378	ExPEC
Gad	100	1,401/1,401	2311375.2312775	Glutamate decarboxylase	AP009378	ExPEC
irp2	100	6,108/6,108	2768325.2774432	High molecular weight protein 2 non-ribosomal peptide synthetase	NZ_NOHG01000003	ExPEC
Iss	97.37	342/342	1923039.1923380	Increased serum survival	AE014075	ExPEC
kpsE	99.48	1,149/1,149	3830099.3831247	Capsule polysaccharide export inner-membrane protein	CP000243	ExPEC
kpsMII_K5	100	777/777	3842521.3843297	Polysialic acid transport protein; Group 2 capsule	UEKQ01000001	ExPEC
nlpI	99.77	886/885	4069831.4070716	Lipoprotein NlpI precursor	CP000243	ExPEC
ompT	93.21	957/954	2209747.2210697	Outer membrane protease (protein protease 7)	NZ_PXZJ01000044	UPEC
ompT	100	954/954	1329943.1330896	Outer membrane protease (protein protease 7)	QODR01000004	UPEC
sitA	100	915/915	1957611.1958525	Iron transport protein	UGBZ01000001ADUG01000044	ExPEC
terC	99.58	714/714	3698355.3699068	Tellurium ion resistance protein	CP000468	ExPEC
terC	97.41	964/966	4004836.4005799	Tellurium ion resistance protein	MG591698	ExPEC
Vat	99.81	4,131/4,131	1073373.1077503	Serine protease autotransporters of Enterobacteriaceae (SPATE)	X16664	ExPEC
yehA	97.58	1,035/1,035	2938665.2939699	Outer membrane lipoprotein, YHD fimbriae l cluster	CP042934	EHEC
yehB	96.61	2,481/2,481	2939715.2942195	Usher, YHD fimbriae l cluster	CP042934	EHEC
yehC	96.89	675/675	2942211.2942885	Chaperone, YHD fimbriae l cluster	CP042934	EHEC
yehD	95.95	543/543	2942966.2943508	Major pilin subunit, YHD fimbriae l cluster	CP042934	EHEC
yfcV	100	567/567	3196771.3197337	Fimbriae l protein	AP018784	ExPEC

% Identity: percent identity in the alignment between the best matching virulence gene in the VirulenceFinder database and the corresponding sequence in the input genome [also called the high-scoring segment pair (HSP)]. A perfect alignment is 100% but must also cover the entire length of the virulence gene in the database; Query/HSP Length: query length is the length of the best matching virulence gene in the database, while HSP length is the length of the alignment between the best matching virulence gene and the corresponding sequence in the genome [also called the high-scoring segment pair (HSP)]; Position in contig: Starting and ending position of the found gene in the contig. Protein function: Known function based on the virulence gene. Accession number: Reference Genbank accession number according to NCBI of the virulence gene in the database; Pathotype: EHEC, enterohemorrhagic; ExPEC, extraintestinal pathogenic; UPEC, uropathogenic; ND, not determined.

A total of two insertion sequences (ISs) and two miniature inverted-repeat transposable elements (MITEs) were grouped into 2 families (ISAs1 and IS630) and were retrieved with the ISfinder web tool. These genomic datasets can provide information on the ecological strength and adaptability of strains as well as their function in various environments. Besides, a total of 399 mobile elements were predicted with mobileOG-db (Brown et al., 2022), from which 50 were insertion/excision, 117 were replication/recombination/repair, 141 belonged to phage, 46 were stability/transfer/defense and 45 were transfer. Based on the 464 matched pathogenic families (10.44% of proteome), the results showed that the L1PEag1 isolate is a human pathogen (0.934 likelihood). Prokka annotation indicated the presence of the *hly*E hemolysis gene in the L1PEag1 genome. The BLASTP analysis indicated a 100% identity to the hemolysin E protein family of the *E. coli* strain AR_451 (a clinical isolate, AWZ82741.1). Hemolysin *hly*E is a novel pore-forming toxin and was first discovered in *E. coli* K-12 (Wyborn et al., 2004). This result was also complemented by the inhibition zone on blood agar media with the effect of hemolytic activity (data not shown). Although gelatinase is a less significant virulence factor in *E. coli* (Shruthi et al., 2012); in the current investigation, the gelatinase enzyme was produced by both L1PEag1 and UTNEc1. These results concur with earlier research, which showed that many *E. coli* isolates from urinary infections were gelatinase positive (Sharma et al., 2007).

### Antibiotic profile

**Figure 3:**
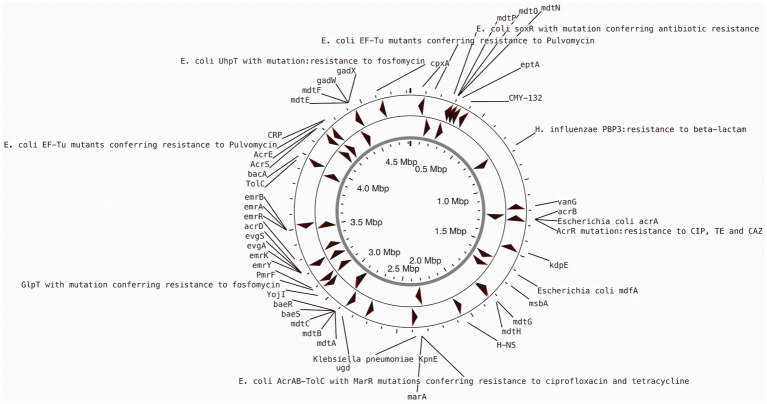
CARD annotation of the antibiotic resistance genes in the L1PEag1 genome.

**Figure 4:**
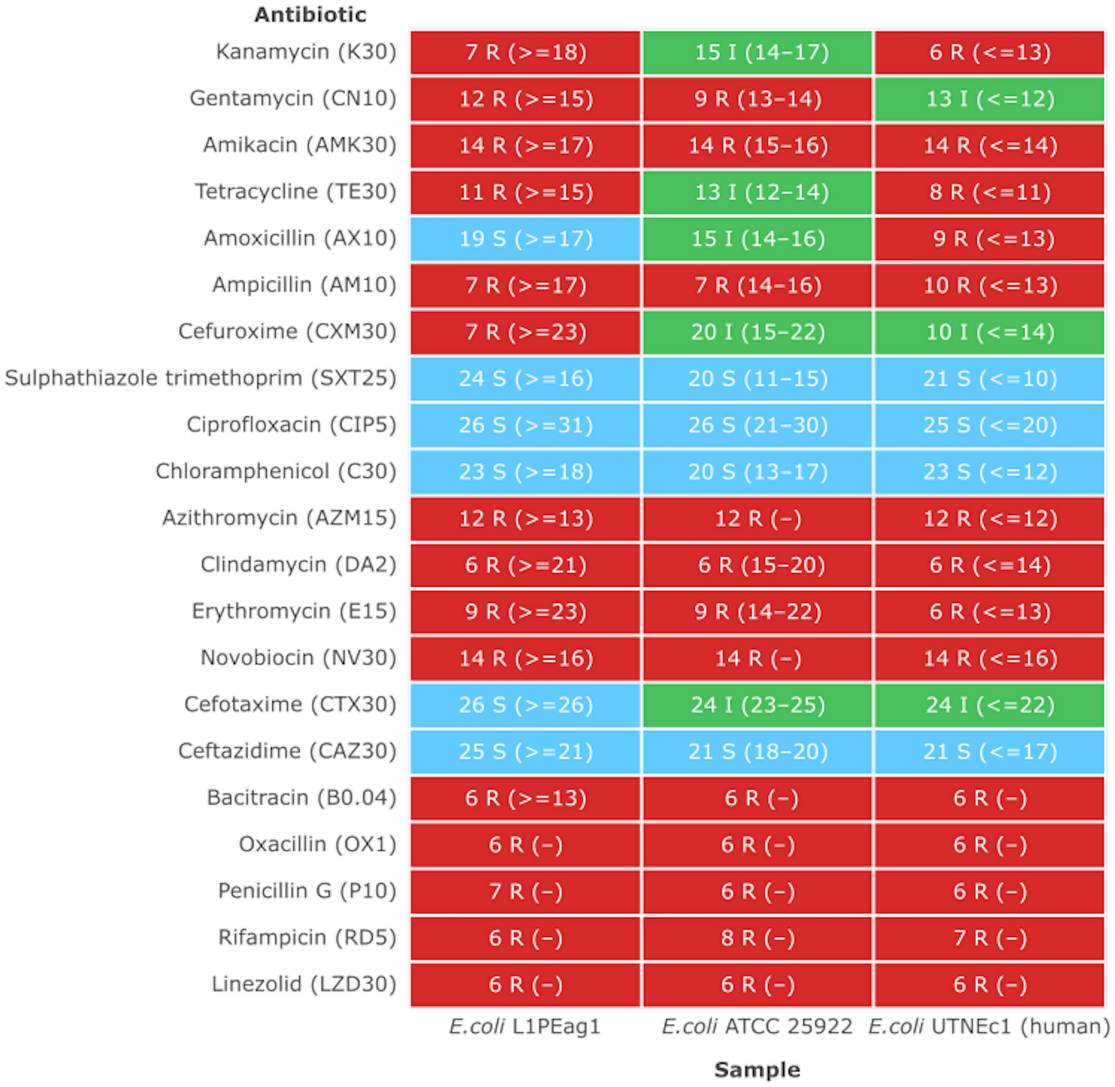
Antimicrobial heatmap profile. The strains showing a MIC higher than the EFSA breakpoint were considered resistant (EFSA Panel on Additives and Products or Substances used in Animal Feed et al., 2018). Susceptible (S, green): a bacterial strain is defined as susceptible when it is inhibited at a concentration of a specific antimicrobial equal to or lower than the established cut-off value (S ≤ x mg/L). Resistant (R, red): a bacterial strain is defined as resistant when it is not inhibited at a concentration of a specific antimicrobial above the established cut-off value (R > x mg/L). Intermediate (I, green): a bacterial strain is defined as intermediate to a given antibiotic when it is inhibited *in vitro* by a concentration of this drug that is associated with an uncertain therapeutic effect. (−) No CLSI standard inhibition zone determined.

Previous studies indicated that *E. coli* is a significant source of resistance genes that could be the cause of treatment failures in veterinary and human medicine. An increasing number of resistance genes have been found in *E. coli* isolates, and many of these resistance genes were acquired through horizontal gene transfer (Poirel et al., 2018). The putative antibiotic resistance genes identified in the genome of L1PEag1 is shown in Figure 3. A total of 4 perfect and 51 strict hits were detected, and the CARD resistant gene identifier report is shown in Supplementary Table S4. Within the former set of hits, the following antibiotic resistance genes were detected *cpx*A, *mdt*H, H-NS, and *evg*A, these confer resistance to aminoglycoside, aminocoumarin, fluoroquinolone and macrolide, cephalosporin, cephamycin, penam, and tetracycline. Previous research in *E. coli* indicated that Cpx-regulated genes are centrally involved in cell energetics, mediated envelope stress adaptation, and antibiotic resistance (Raivio et al., 2013). The *mdt*H (multidrug transport) gene is one of the 35 efflux pumping encoding genes detected in *E. coli.* Overexpression of cloned *mdt*D in the *E. coli* K-12 strain, which lacks the major efflux pump permease AcrB (*E. coli* KAM3) (Hirakawa et al., 2003a), results in a two-fold increase in resistance to norfloxacin and enoxacin (compared to the mutant parent) but does not impact the resistance to a range of other toxic compounds (dyes, detergents, antibiotics, and others) (Nishino and Yamaguchi, 2001). H-NS is a histone-like protein involved in global gene regulation in Gram-negative bacteria (Nishino and Yamaguchi, 2004). It is a repressor of the membrane fusion protein genes *acr*E, *mdt*E, and *emr*K as well as nearby genes of many intrinsic multidrug exporters (Nishino and Yamaguchi, 2004). EvgA, when phosphorylated, is a positive regulator for efflux protein complexes emrKY and mdtEF (Hirakawa et al., 2003b) which confer resistance to erythromycin, cloxacillin, tetracycline, oxacillin, and norfloxacin. Notably, an SNP mutation (R234F) was found in the *E. coli* elongation factor EF-Tu, which confers resistance to pulvomycin. The *E. coli glp*T gene (E448K) confers resistance to fosfomycin. The UhpT (E350Q) carries a mutation that confers resistance to fosfomycin; The PBP3 (D350N, S357N) gene, similar to *Haemophilus influenzae*, carries a mutation that confers resistance to beta-lactam antibiotics; and *E. coli* AcrAB-TolC bears MarR mutations (Y137H, G103S), which confer resistance to ciprofloxacin and tetracycline (Supplementary Table S4). The resistance to azithromycin (AZM15), clindamycin (DA2), novobiocin (NV30), amikacin (AMK30), oxacillin (OX1), erythromycin (E15), bacitracin, and other antibiotics was confirmed by the disk assay method; out of the 21 antibiotics tested, the target strain showed resistance to 14 antibiotics belonging to several classes as shown in Figure 4. Antibiotic resistance was previously evaluated in several *E. coli* strains isolated from lettuce, cabbage, cucumbers, and tomatoes (Rahman et al., 2021). Besides, 100% of the *E. coli* isolates obtained from fruits and vegetables collected from various regions in Ecuador, exhibited resistance to ampicillin, cefazolin, cefotaxime, and tetracycline (Montero et al., 2021). The strains isolated from fresh produce have demonstrated variable resistance to colistin (CST), AMK, E15, cefotaxime (CTX), ceftazidime (CAZ), AMP, gentamycin (GEN), and amoxicillin (AMX) (Shah et al., 2015; Gómez-Aldapa et al., 2016). In addition, *E. coli* isolated from fresh vegetables (i.e., pak choi and lettuce) were resistant to kanamycin, levofloxacin, doxycycline, fosfomycin (FOS), CTX, AMP, GEN, NA, TE, and colistin (CST) (Liu B-T et al., 2019; Manageiro et al., 2020). PCR analysis results confirmed the resistance to tetracycline (*bla*_TEM-1_) and carbapenemase (*bla*_KPC_) (Supplementary Table S1). The gene bla_TEM-1_ is widely found in animal-derived *E. coli* and encodes for β-lactamases with a narrow spectrum that are capable of inactivating aminopenicillins and penicillins (Poirel et al., 2018). In the study of Montero et al. (2021) there were differences in a subset of ESBL-*E. coli* isolates that tested positive for certain genes by PCR but negative by whole genome sequencing (WGS); these were 12 isolates with blaTEM, nine isolates with blaSHV, and one isolate with blaCTX-M. Furthermore, WGS revealed blaSHV and blaTEM in two isolates, but PCR results were negative. A recent study revealed a relatively high prevalence of *bla*_KPC_–carrying, carbapenemase-resistant *Enterobacteriaceae* in fecal isolates, among food handlers in Kuwait (Moghnia et al., 2021). Nonetheless, non-specific amplification products were detected for *bla*_VIM_ (Verona integron-encoded metallo-β-lactamase), oxacillinases (OXA48/181), and cefotaxime (*bla*_CTXM-9_). This might be produced by primers binding to seemingly random locations in the sample DNA. No amplification was detected for the other tested β-lactam genes (Supplementary Table S1).

Based on RGI analysis, the *sit*ABCD genes were detected in the genome of the L1PEag1 isolate (i.e., *sit*A: a periplasmic binding protein, *sit*B: an ATP-binding component, *sit*C: an inner membrane component, and *sit*D: an inner membrane component, which conferred resistance to hydrogen peroxide). Along with other ion transport systems, SitABCD is an iron and manganese transporter that may confer oxidative stress resistance and bactericidal effects. These genes showed 97.31% identity with a sitABCD gene located on the virulence plasmid pAPEC-1 of an avian pathogenic *E. coli* strain (AY598030.2) (Sabri et al., 2006).

## Conclusion

To the best of our knowledge, this is the first genome characterization of an *E. coli* isolate L1PEag1, originating from cape gooseberry ready-to-eat fruit, and that also shows antibiotic resistance. The L1PEag1 isolate belongs to the B2 phylogroup, sequence type ST1170, and serotype O1:H4 based on *in silico* genome analysis. L1PEag1 genome harbored several virulence factors and was predicted as a human pathogen. Based on the results we speculate that this isolate could cross contaminate the fruit at the studied market sites. It illustrates the possible contribution of the fruit environment to the spread of pathogenic *E. coli* strains. The potential risks to public health posed by the presence of antibiotic resistance genes in ready-to-eat cape gooseberry are unknown. This emphasizes the need for a comprehensive strategy that addresses pre- and post-harvest processing as well as produce distribution. Our work emphasizes the necessity of monitoring environmental antibiotic resistance, particularly in commercial settings, since this would help in developing early mitigation techniques for dealing with resistance diffusion.

## Data availability statement

The datasets presented in this study can be found in online repositories. The names of the repository/repositories and accession number(s) can be found in the article/Supplementary material.

## Author contributions

DM: Investigation, Writing – review & editing. JCC-O: Writing – review & editing. PJ-V: Software, Writing – review & editing. GNT: Conceptualization, Data curation, Formal analysis, Funding acquisition, Investigation, Methodology, Project administration, Resources, Software, Supervision, Validation, Writing – original draft, Writing – review & editing.

## Footnotes

^1^ http://www.bioinformatics.babraham.ac.uk/projects/fastqc

^2^ https://github.com/nanoporetech/medaka

^3^ http://www.ezclermont.org

^4^ https://cge.food.dtu.dk/services/MLST

^5^ https://cge.food.dtu.dk/services/SerotypeFinder

^6^ http://www.cbs.dtu.dk/services/RNAmmer

^7^ http://www.ansikte.se/ARAGORN

^8^ http://www.cbs.dtu.dk/services/SignalP

^9^ http://gctype.wdcm.org

^10^ https://www.uniprot.org/statistics/Swiss-Prot

^11^ https://metacyc.org

^12^ http://www.mgc.ac.cn/VFs/main.htm

^13^ http://www.phi-base.org/searchFacet.htm?queryTerm

^14^ http://xfam.org

^15^ https://proksee.ca/projects/new

^16^ https://crisprcas.i2bc.paris-saclay.fr/CrisprCasFinder/Index

^17^ http://phaster.ca

^18^ http://arpcard.mcmaster.ca

^19^ https://cge.cbs.dtu.dk/services/ResFinder/

^20^ https://cge.food.dtu.dk/services/VirulenceFinder/

^21^ http://cge.cbs.dtu.dk/services/PathogenFinder/

^22^ https://cge.food.dtu.dk/services/FimTyper/

^23^ https://www.normalizacion.gob.ec/buzon/normas/1529–2-1R.pdf

## Supplementary material

SUPPLEMENTARY FIGURE S1 Mapping of the COG **(A)** and KEGG **(B)** proteins according to their function. The number of genes in each category is shown.
