# Supplementary material for: Genome characterization of a multi-drug resistant Escherichia coli strain, L1PEag1, isolated from commercial cape gooseberry fruits (Physalis peruviana L.)
Source: Front Microbiol. 2024 Jul 22;15:1392333. doi: 10.3389/fmicb.2024.1392333 (PMC11298459; doi:10.3389/fmicb.2024.1392333)
Supplement: SUPPLEMENTARY FIGURE S1 — Mapping of the COG (A) and KEGG (B) proteins according to their function. The number of genes in each category is shown. [file Table_1.DOCX]

Supplementary Material

**Genome characterization of a multi-drug resistant *Escherichia coli* strain, L1PEag1, isolated from commercial cape gooseberry fruits (*Physalis peruviana* L*.)***

Diana Molina^1^, Julio C. Carrión–Olmedo^2^, Pablo Jarrín–V.^1,2^ and Gabriela N. Tenea^1^*

^1^Biofood and Nutraceutics Research and Development Group; Faculty of Engineering in Agricultural and Environmental Sciences, Universidad Técnica del Norte, 100150 Ibarra, Ecuador.

^2^Laboratorio de Secuenciamiento de Ácidos Nucleicos, Dirección de Innovación, Instituto Nacional de Biodiversidad (INABIO), 170506 Quito, Ecuador.

*Correspondence:

Corresponding author: Gabriela N. Tenea

gntenea@utn.edu.ec

**Supplementary Table 1.** List of genes and primers for *ß*-lactam resistance and virulence determinants genes and the PCR results detection in L1PEag1, *E. coli* ATCC25992 and *E. coli* UTNEc1 (human).

| **Gene** | **Lactamase class/ antibiotic** | **Primer’s name** | **Sequence (5’-3’)** | **PCR product (bp)** | **L1PEag1** | ***E. coli* ATCC25922** | ***E. coli* UTNEc1 (human)** |
| --- | --- | --- | --- | --- | --- | --- | --- |
| *bla*_TEM-1_ | Class A/ Temoniera-lactamase | TEM-410FW | GGTCGCCGCATACACTATTCTC | 372 | + | + | + |
|  |  | TEM-781RW | TTTATCCGCCTCCATCCAGTC |  |  |  |  |
| *bla*_SHV_ | Class A/ Sulfhydryl variable-lactamase | SHV-287FW | CCAGCAGGATCTGGTGGACTA | 231 | - | - | - |
|  |  | SHV-517RW | CCGGGAAGCGCCTCAT |  |  |  |  |
| *bla*_CTXM-2_ | Class A/ Cefotaximase-München-lactamase | ctxm2-39FW | GATGGCGACGCTACCCC | 107 | - | - | - |
|  |  | ctxm2-145RW | CAAGCCGACCTCCCGAAC |  |  |  |  |
| *bla*_CTXM-8/25_ | Class A/ Cefotaximase-München-lactamase | ctxm8g25g-533FW | GCGACCCGCGCGATAC | 186 | ** | ** | ** |
|  |  | ctxm8g25g-718RW | TGCCGGTTTTATCCCCG |  |  |  |  |
| *bla*_CTXM-9_ | Class A/ Cefotaximase-München-lactamase | ctxm9-16FW | GTGCAACGGATGATGTTCGC | 475 | ** | ** | ** |
|  |  | ctxm9-490RW | GAAACGTCTCATCGCCGATC |  |  |  |  |
| *bla*_KPC_ | Class A/ *Klebsiella pneumoniae* carbapenemase | KPC-RW | CGTCTAGTTCTGCTGTCTTG | 798 | + | + | + |
|  |  | KPC-RW | CTTGTCATCCTTGTTAGGCG |  |  |  |  |
| *bla*_VIM_ | Class B/ Verona integron-encoded metallo-β-lactamase | VIM-FW | GATGGTGTTTGGTCGCATA | 390 | ** | - | - |
|  |  | VIM-RW | CGAATGCGCAGCACCAG |  |  |  |  |
| *bla*_NDM_ | Class B/ New Delhi metallo-β-lactamase | NDM-FW | GGTTTGGCGATCTGGTTTTC | 621 | - | - | - |
|  |  | NDM-RW | CGGAATGGCTCATCACGATC |  |  |  |  |
| *bla*_OXA-48/181_ | Class D/ oxacillinases | OXA48-FW | GCGTGGTTAAGGATGAACAC | 438 | ** | ** | ** |
|  |  | OXA48-RW | CATCAAGTTCAACCCAACCG |  |  |  |  |
| *fimH* | Type I fibrinae | fimH-FW | AACAGCGATGATTTCCAGTTTGTGTG | 465 | + | + | + |
|  |  | fimH.RW | ATTGCGTACCAGCATTAGCAATGTCC |  |  |  |  |
| *papC* | pili associated with pyelonephritis | Pap1 | GACGGCTGTACTGCAGGGTGTGGCG | 328 | + | + | + |
|  |  | Pap2 | ATATCCTTTCTGCAGGGATGCAATA |  |  |  |  |
| *sfa* | S fibrinae | Sfa1 | CTCCGGAGAACTGGGTGCATCTTAC | 410 | - | + | + |
|  |  | Sfa2 | CGGAGGAGTAATTACAAACCTGGCA |  |  |  |  |

+: amplicon detected; - no amplification. **- non-specific amplification

**Supplementary Table 2.** Sequence type, serotype, and virulence genes.

| **Sample** | **ST/ nearest ST** | **Serotype** | **Locus** | **Identity** | **Coverage** | **Alignment Length** | **Allele Length** | **Gaps** | **Allele** |
| --- | --- | --- | --- | --- | --- | --- | --- | --- | --- |
| L1PEag1 | ST1170 | O1-H4 | Adk | 100 | 100 | 536 | 536 | 0 | adk_13 |
|  | ST1170 | O1-H4 | fumC | 100 | 100 | 469 | 469 | 0 | fumC_40 |
|  | ST1170 | O1-H4 | gyrB | 100 | 100 | 460 | 460 | 0 | gyrB_19 |
|  | ST1170 | O1-H4 | Icd | 100 | 100 | 518 | 518 | 0 | icd_13 |
|  | ST1170 | O1-H4 | mdh | 100 | 100 | 452 | 452 | 0 | mdh_23 |
|  | ST1170 | O1-H4 | purA | 100 | 100 | 478 | 478 | 0 | purA_28 |
|  | ST1170 | O1-H4 | recA | 100 | 100 | 510 | 510 | 0 | recA_109 |
|  | ST518,966 | O1-H4 | dinB | 100 | 100 | 450 | 450 | 0 | dinB_88 |
|  | ST518,966 | O1-H4 | icdA | 100 | 100 | 516 | 516 | 0 | icdA_15 |
|  | ST518,966 | O1-H4 | pabB | 100 | 100 | 468 | 468 | 0 | pabB_127 |
|  | ST518,966 | O1-H4 | polB | 100 | 100 | 450 | 450 | 0 | polB_76 |
|  | ST518,966 | O1-H4 | putP | 100 | 100 | 456 | 456 | 0 | putP_4 |
|  | ST518,966 | O1-H4 | trpA | 100 | 100 | 561 | 561 | 0 | trpA_72 |
|  | ST518,966 | O1-H4 | trpB | 100 | 100 | 594 | 594 | 0 | trpB_1 |
|  | ST518,966 | O1-H4 | uidA | 100 | 100 | 600 | 600 | 0 | uidA_9 |

**Supplementary Table 3.** *In silico* island picks detected in the L1PEag1 genome.

| **Island end** | **Length** | **Method** | **Gene ID** | **Locus** | **Gene start** | **Gene end** | **Strand** | **Product** |
| --- | --- | --- | --- | --- | --- | --- | --- | --- |
| 361007 | 13213 | IslandPick | fimD_1 | MACIJPLA_00316 | 347903 | 350440 | -1 | Outer membrane usher protein FimD |
| 361007 | 13213 | IslandPick | yraI | MACIJPLA_00317 | 350468 | 351172 | -1 | putative fimbrial chaperone YraI |
| 361007 | 13213 | IslandPick |  | MACIJPLA_00318 | 351234 | 351803 | -1 | hypothetical protein |
| 361007 | 13213 | IslandPick | Tsh | MACIJPLA_00319 | 352531 | 356070 | 1 | Temperature-sensitive hemagglutinin tsh autotransporter |
| 361007 | 13213 | IslandPick |  | MACIJPLA_00320 | 356277 | 356759 | 1 | hypothetical protein |
| 361007 | 13213 | IslandPick | virF | MACIJPLA_00321 | 357007 | 357798 | 1 | Virulence regulon transcriptional activator VirF |
| 361007 | 13213 | IslandPick |  | MACIJPLA_00322 | 357919 | 358098 | -1 | hypothetical protein |
| 361007 | 13213 | IslandPick | espC | MACIJPLA_00323 | 358953 | 362882 | 1 | Serine protease EspC |
| 635373 | 5001 | IslandPick |  | MACIJPLA_00596 | 630274 | 630873 | 1 | hypothetical protein |
| 635373 | 5001 | IslandPick |  | MACIJPLA_00597 | 630870 | 631418 | 1 | hypothetical protein |
| 635373 | 5001 | IslandPick |  | MACIJPLA_00598 | 631479 | 634892 | 1 | hypothetical protein |
| 635373 | 5001 | IslandPick | ompX_1 | MACIJPLA_00599 | 634963 | 635562 | 1 | Outer membrane protein X |
| 994882 | 26096 | IslandPick | fliP_1 | MACIJPLA_00899 | 968416 | 969168 | -1 | Flagellar biosynthetic protein FliP |
| 994882 | 26096 | IslandPick |  | MACIJPLA_00900 | 969165 | 969536 | -1 | hypothetical protein |
| 994882 | 26096 | IslandPick |  | MACIJPLA_00901 | 969529 | 970380 | -1 | hypothetical protein |
| 994882 | 26096 | IslandPick | vnfA | MACIJPLA_00902 | 970767 | 971753 | 1 | Nitrogen fixation protein VnfA |
| 994882 | 26096 | IslandPick | fliE_1 | MACIJPLA_00903 | 971768 | 972109 | 1 | Flagellar hook-basal body complex protein FliE |
| 994882 | 26096 | IslandPick | fliF_1 | MACIJPLA_00904 | 972114 | 973760 | 1 | Flagellar M-ring protein |
| 994882 | 26096 | IslandPick | fliG_1 | MACIJPLA_00905 | 973738 | 974748 | 1 | Flagellar motor switch protein FliG |
| 994882 | 26096 | IslandPick |  | MACIJPLA_00906 | 974752 | 975462 | 1 | hypothetical protein |
| 994882 | 26096 | IslandPick | fliI_1 | MACIJPLA_00907 | 975455 | 976795 | 1 | Flagellum-specific ATP synthase |
| 994882 | 26096 | IslandPick |  | MACIJPLA_00908 | 976798 | 977232 | 1 | hypothetical protein |
| 994882 | 26096 | IslandPick | tagD | MACIJPLA_00909 | 977235 | 977639 | 1 | Glycerol-3-phosphate cytidylyltransferase |
| 994882 | 26096 | IslandPick | fic_1 | MACIJPLA_00910 | 977633 | 978709 | -1 | Protein adenylyltransferase SoFic |
| 994882 | 26096 | IslandPick |  | MACIJPLA_00911 | 978990 | 979199 | 1 | hypothetical protein |
| 994882 | 26096 | IslandPick |  | MACIJPLA_00912 | 979225 | 979935 | -1 | hypothetical protein |
| 994882 | 26096 | IslandPick |  | MACIJPLA_00913 | 980038 | 980682 | -1 | hypothetical protein |
| 994882 | 26096 | IslandPick |  | MACIJPLA_00914 | 981110 | 983548 | -1 | hypothetical protein |
| 994882 | 26096 | IslandPick |  | MACIJPLA_00915 | 983595 | 984527 | -1 | hypothetical protein |
| 994882 | 26096 | IslandPick |  | MACIJPLA_00916 | 984655 | 985083 | -1 | hypothetical protein |
| 994882 | 26096 | IslandPick |  | MACIJPLA_00917 | 985096 | 985374 | -1 | hypothetical protein |
| 994882 | 26096 | IslandPick |  | MACIJPLA_00918 | 985456 | 986193 | -1 | hypothetical protein |
| 994882 | 26096 | IslandPick | flgB_1 | MACIJPLA_00919 | 986275 | 986610 | 1 | Flagellar basal body rod protein FlgB |
| 994882 | 26096 | IslandPick | flgC_1 | MACIJPLA_00920 | 986613 | 987044 | 1 | Flagellar basal-body rod protein FlgC |
| 994882 | 26096 | IslandPick |  | MACIJPLA_00921 | 987044 | 987757 | 1 | hypothetical protein |
| 994882 | 26096 | IslandPick |  | MACIJPLA_00922 | 987757 | 987912 | 1 | hypothetical protein |
| 994882 | 26096 | IslandPick | flgE_1 | MACIJPLA_00923 | 987925 | 989127 | 1 | Flagellar hook protein FlgE |
| 994882 | 26096 | IslandPick | flgF_1 | MACIJPLA_00924 | 989127 | 989864 | 1 | Flagellar basal-body rod protein FlgF |
| 994882 | 26096 | IslandPick | flgG_1 | MACIJPLA_00925 | 989943 | 990728 | 1 | Flagellar basal-body rod protein FlgG |
| 994882 | 26096 | IslandPick | flgH_1 | MACIJPLA_00926 | 990736 | 991476 | 1 | Flagellar L-ring protein |
| 994882 | 26096 | IslandPick | flgI_1 | MACIJPLA_00927 | 991476 | 992591 | 1 | Flagellar P-ring protein |
| 994882 | 26096 | IslandPick |  | MACIJPLA_00928 | 992591 | 992890 | 1 | hypothetical protein |
| 994882 | 26096 | IslandPick |  | MACIJPLA_00929 | 993080 | 994456 | 1 | hypothetical protein |
| 994882 | 26096 | IslandPick | flgL_1 | MACIJPLA_00930 | 994471 | 995400 | 1 | Flagellar hook-associated protein 3 |
| 999764 | 4865 | IslandPick | flgL_1 | MACIJPLA_00930 | 994471 | 995400 | 1 | Flagellar hook-associated protein 3 |
| 999764 | 4865 | IslandPick |  | MACIJPLA_00931 | 995417 | 996394 | 1 | hypothetical protein |
| 999764 | 4865 | IslandPick |  | MACIJPLA_00932 | 996453 | 997295 | -1 | hypothetical protein |
| 999764 | 4865 | IslandPick | fliC1 | MACIJPLA_00933 | 997781 | 998695 | 1 | Flagellin 1 |
| 999764 | 4865 | IslandPick | fliD_1 | MACIJPLA_00934 | 999105 | 1000421 | 1 | Flagellar hook-associated protein 2 |
| 1049849 | 13234 | IslandPick |  | MACIJPLA_00986 | 1036207 | 1037448 | 1 | hypothetical protein |
| 1049849 | 13234 | IslandPick |  | MACIJPLA_00987 | 1037426 | 1038076 | 1 | hypothetical protein |
| 1049849 | 13234 | IslandPick |  | MACIJPLA_00988 | 1038091 | 1039296 | 1 | hypothetical protein |
| 1049849 | 13234 | IslandPick |  | MACIJPLA_00989 | 1039346 | 1039546 | 1 | hypothetical protein |
| 1049849 | 13234 | IslandPick |  | MACIJPLA_00990 | 1039549 | 1039872 | 1 | hypothetical protein |
| 1049849 | 13234 | IslandPick |  | MACIJPLA_00991 | 1039869 | 1040279 | 1 | hypothetical protein |
| 1049849 | 13234 | IslandPick |  | MACIJPLA_00992 | 1040254 | 1040760 | 1 | hypothetical protein |
| 1049849 | 13234 | IslandPick |  | MACIJPLA_00993 | 1040757 | 1041317 | 1 | hypothetical protein |
| 1049849 | 13234 | IslandPick |  | MACIJPLA_00994 | 1041326 | 1041496 | 1 | hypothetical protein |
| 1049849 | 13234 | IslandPick |  | MACIJPLA_00995 | 1041480 | 1042976 | 1 | hypothetical protein |
| 1049849 | 13234 | IslandPick |  | MACIJPLA_00996 | 1042976 | 1043332 | 1 | hypothetical protein |
| 1049849 | 13234 | IslandPick |  | MACIJPLA_00997 | 1043332 | 1043601 | 1 | hypothetical protein |
| 1049849 | 13234 | IslandPick |  | MACIJPLA_00998 | 1043743 | 1045575 | 1 | hypothetical protein |
| 1049849 | 13234 | IslandPick |  | MACIJPLA_00999 | 1045667 | 1046197 | 1 | hypothetical protein |
| 1049849 | 13234 | IslandPick |  | MACIJPLA_01000 | 1046259 | 1047587 | 1 | hypothetical protein |
| 1049849 | 13234 | IslandPick |  | MACIJPLA_01001 | 1047584 | 1048663 | 1 | hypothetical protein |
| 1049849 | 13234 | IslandPick |  | MACIJPLA_01002 | 1048663 | 1049211 | 1 | hypothetical protein |
| 1049849 | 13234 | IslandPick |  | MACIJPLA_01003 | 1049211 | 1049636 | 1 | hypothetical protein |
| 1049849 | 13234 | IslandPick |  | MACIJPLA_01004 | 1049623 | 1050681 | 1 | hypothetical protein |
| 1117429 | 5988 | IslandPick |  | MACIJPLA_01059 | 1111516 | 1113804 | -1 | hypothetical protein |
| 1117429 | 5988 | IslandPick |  | MACIJPLA_01060 | 1113846 | 1114607 | -1 | hypothetical protein |
| 1117429 | 5988 | IslandPick |  | MACIJPLA_01061 | 1114624 | 1114764 | -1 | hypothetical protein |
| 1117429 | 5988 | IslandPick |  | MACIJPLA_01062 | 1114761 | 1115555 | -1 | hypothetical protein |
| 1117429 | 5988 | IslandPick |  | MACIJPLA_01063 | 1115885 | 1116448 | -1 | ISKra4 family transposase ISCep1 |
| 1117429 | 5988 | IslandPick |  | MACIJPLA_01064 | 1117208 | 1117489 | 1 | hypothetical protein |
| 1913374 | 6828 | IslandPick |  | MACIJPLA_01800 | 1906268 | 1906948 | -1 | hypothetical protein |
| 1913374 | 6828 | IslandPick |  | MACIJPLA_01801 | 1906945 | 1907730 | -1 | hypothetical protein |
| 1913374 | 6828 | IslandPick |  | MACIJPLA_01802 | 1907736 | 1908032 | -1 | hypothetical protein |
| 1913374 | 6828 | IslandPick |  | MACIJPLA_01803 | 1908108 | 1908314 | -1 | hypothetical protein |
| 1913374 | 6828 | IslandPick |  | MACIJPLA_01804 | 1908787 | 1909236 | -1 | hypothetical protein |
| 1913374 | 6828 | IslandPick |  | MACIJPLA_01805 | 1909217 | 1910242 | -1 | hypothetical protein |
| 1913374 | 6828 | IslandPick |  | MACIJPLA_01806 | 1910336 | 1911025 | -1 | putative HTH-type transcriptional regulator |
| 1913374 | 6828 | IslandPick |  | MACIJPLA_01807 | 1911130 | 1911360 | 1 | hypothetical protein |
| 1913374 | 6828 | IslandPick |  | MACIJPLA_01808 | 1911430 | 1911969 | 1 | hypothetical protein |
| 1913374 | 6828 | IslandPick |  | MACIJPLA_01809 | 1911981 | 1912985 | 1 | hypothetical protein |
| 1913374 | 6828 | IslandPick |  | MACIJPLA_01810 | 1912982 | 1913683 | 1 | hypothetical protein |
| 1951883 | 4011 | IslandPick |  | MACIJPLA_01857 | 1947564 | 1948136 | 1 | hypothetical protein |
| 1951883 | 4011 | IslandPick |  | MACIJPLA_01858 | 1948196 | 1951609 | 1 | hypothetical protein |
| 1951883 | 4011 | IslandPick | Ail | MACIJPLA_01859 | 1951679 | 1952278 | 1 | Attachment invasion locus protein |
| 2190767 | 11564 | IslandPick |  | MACIJPLA_02095 | 2178951 | 2179742 | 1 | hypothetical protein |
| 2190767 | 11564 | IslandPick |  | MACIJPLA_02096 | 2179735 | 2180667 | 1 | hypothetical protein |
| 2190767 | 11564 | IslandPick |  | MACIJPLA_02097 | 2180645 | 2180854 | 1 | hypothetical protein |
| 2190767 | 11564 | IslandPick |  | MACIJPLA_02098 | 2180858 | 2181952 | 1 | hypothetical protein |
| 2190767 | 11564 | IslandPick |  | MACIJPLA_02099 | 2181933 | 2183234 | 1 | hypothetical protein |
| 2190767 | 11564 | IslandPick |  | MACIJPLA_02100 | 2183237 | 2184643 | 1 | hypothetical protein |
| 2190767 | 11564 | IslandPick |  | MACIJPLA_02101 | 2184624 | 2185739 | 1 | hypothetical protein |
| 2190767 | 11564 | IslandPick |  | MACIJPLA_02102 | 2185844 | 2186608 | 1 | hypothetical protein |
| 2190767 | 11564 | IslandPick |  | MACIJPLA_02103 | 2186707 | 2187846 | 1 | hypothetical protein |
| 2190767 | 11564 | IslandPick |  | MACIJPLA_02104 | 2187889 | 2188065 | 1 | hypothetical protein |
| 2190767 | 11564 | IslandPick |  | MACIJPLA_02105 | 2188069 | 2188464 | 1 | hypothetical protein |
| 2190767 | 11564 | IslandPick |  | MACIJPLA_02106 | 2188464 | 2188847 | 1 | hypothetical protein |
| 2190767 | 11564 | IslandPick |  | MACIJPLA_02107 | 2188848 | 2189228 | 1 | hypothetical protein |
| 2190767 | 11564 | IslandPick |  | MACIJPLA_02108 | 2189225 | 2189617 | 1 | hypothetical protein |
| 2190767 | 11564 | IslandPick |  | MACIJPLA_02109 | 2189644 | 2190606 | 1 | hypothetical protein |
| 2190767 | 11564 | IslandPick |  | MACIJPLA_02110 | 2190667 | 2191116 | 1 | hypothetical protein |
| 2211326 | 7260 | IslandPick |  | MACIJPLA_02120 | 2204022 | 2205062 | 1 | hypothetical protein |
| 2211326 | 7260 | IslandPick |  | MACIJPLA_02121 | 2205105 | 2205395 | 1 | hypothetical protein |
| 2211326 | 7260 | IslandPick |  | MACIJPLA_02122 | 2205908 | 2206621 | 1 | hypothetical protein |
| 2211326 | 7260 | IslandPick | cdtB | MACIJPLA_02123 | 2206618 | 2207439 | 1 | Cytolethal distending toxin subunit B |
| 2211326 | 7260 | IslandPick |  | MACIJPLA_02124 | 2207436 | 2208008 | 1 | hypothetical protein |
| 2211326 | 7260 | IslandPick | caeB | MACIJPLA_02125 | 2208078 | 2209562 | -1 | Carboxylesterase B |
| 2211326 | 7260 | IslandPick | ompT_2 | MACIJPLA_02126 | 2209747 | 2210697 | -1 | Protease 7 |
| 2755639 | 9546 | IslandPick |  | MACIJPLA_02646 | 2746253 | 2746726 | 1 | hypothetical protein |
| 2755639 | 9546 | IslandPick |  | MACIJPLA_02647 | 2746758 | 2746970 | 1 | hypothetical protein |
| 2755639 | 9546 | IslandPick |  | MACIJPLA_02648 | 2746967 | 2747134 | 1 | hypothetical protein |
| 2755639 | 9546 | IslandPick |  | MACIJPLA_02649 | 2747219 | 2748241 | -1 | hypothetical protein |
| 2755639 | 9546 | IslandPick |  | MACIJPLA_02650 | 2748290 | 2748430 | -1 | hypothetical protein |
| 2755639 | 9546 | IslandPick |  | MACIJPLA_02651 | 2748486 | 2748872 | -1 | hypothetical protein |
| 2755639 | 9546 | IslandPick |  | MACIJPLA_02652 | 2748955 | 2749224 | -1 | hypothetical protein |
| 2755639 | 9546 | IslandPick |  | MACIJPLA_02653 | 2749407 | 2749505 | -1 | hypothetical protein |
| 2755639 | 9546 | IslandPick |  | MACIJPLA_02654 | 2750102 | 2750836 | 1 | hypothetical protein |
| 2755639 | 9546 | IslandPick |  | MACIJPLA_02655 | 2750861 | 2751652 | 1 | hypothetical protein |
| 2755639 | 9546 | IslandPick |  | MACIJPLA_02656 | 2751738 | 2752217 | 1 | hypothetical protein |
| 2755639 | 9546 | IslandPick | hns_2 | MACIJPLA_02657 | 2752373 | 2752777 | 1 | DNA-binding protein H-NS |
| 2755639 | 9546 | IslandPick |  | MACIJPLA_02658 | 2753224 | 2754597 | 1 | hypothetical protein |
| 2755639 | 9546 | IslandPick |  | MACIJPLA_02659 | 2755016 | 2755405 | -1 | hypothetical protein |
| 2755639 | 9546 | IslandPick |  | MACIJPLA_02660 | 2755455 | 2755697 | -1 | hypothetical protein |
| 2776374 | 17178 | IslandPick | intA_4 | MACIJPLA_02668 | 2759575 | 2760618 | 1 | Prophage integrase IntA |
| 2776374 | 17178 | IslandPick | mbtI | MACIJPLA_02669 | 2760812 | 2762116 | -1 | Salicylate synthase |
| 2776374 | 17178 | IslandPick | ampG_2 | MACIJPLA_02670 | 2762144 | 2763424 | -1 | Anhydromuropeptide permease |
| 2776374 | 17178 | IslandPick |  | MACIJPLA_02671 | 2763417 | 2765219 | -1 | Putative multidrug export ATP-binding/permease protein |
| 2776374 | 17178 | IslandPick | btuD_4 | MACIJPLA_02672 | 2765206 | 2767008 | -1 | Vitamin B12 import ATP-binding protein BtuD |
| 2776374 | 17178 | IslandPick |  | MACIJPLA_02673 | 2767175 | 2768134 | 1 | hypothetical protein |
| 2776374 | 17178 | IslandPick | dltA | MACIJPLA_02674 | 2768325 | 2774432 | 1 | D-alanine--D-alanyl carrier protein ligase |
| 2776374 | 17178 | IslandPick | COQ5_2 | MACIJPLA_02675 | 2774520 | 2784011 | 1 | 2-methoxy-6-polyprenyl-1,4-benzoquinol methylase, mitocondrial |
| 2790649 | 9279 | IslandPick | COQ5_2 | MACIJPLA_02675 | 2774520 | 2784011 | 1 | 2-methoxy-6-polyprenyl-1,4-benzoquinol methylase, mitocondrial |
| 2790649 | 9279 | IslandPick |  | MACIJPLA_02676 | 2784008 | 2785108 | 1 | hypothetical protein |
| 2790649 | 9279 | IslandPick | pikAV | MACIJPLA_02677 | 2785105 | 2785908 | 1 | Thioesterase PikA5 |
| 2790649 | 9279 | IslandPick | dhbE | MACIJPLA_02678 | 2785912 | 2787489 | 1 | 2,3-dihydroxybenzoate-AMP ligase |
| 2790649 | 9279 | IslandPick | fyuA | MACIJPLA_02679 | 2787621 | 2789642 | 1 | Pesticin receptor |
| 2790649 | 9279 | IslandPick |  | MACIJPLA_02680 | 2790294 | 2791043 | 1 | hypothetical protein |
| 2812328 | 5934 | IslandPick | gatC_1 | MACIJPLA_02696 | 2805468 | 2806721 | -1 | PTS system galactitol-specific EIIC component |
| 2812328 | 5934 | IslandPick |  | MACIJPLA_02697 | 2806742 | 2807035 | -1 | hypothetical protein |
| 2812328 | 5934 | IslandPick | gatA_1 | MACIJPLA_02698 | 2807054 | 2807497 | -1 | PTS system galactitol-specific EIIA component |
| 2812328 | 5934 | IslandPick | ydjF_2 | MACIJPLA_02699 | 2807836 | 2808591 | -1 | putative HTH-type transcriptional regulator YdjF |
| 2812328 | 5934 | IslandPick | lacB | MACIJPLA_02700 | 2808940 | 2809476 | 1 | Galactose-6-phosphate isomerase subunit LacB |
| 2812328 | 5934 | IslandPick | gatC_2 | MACIJPLA_02701 | 2809480 | 2810850 | 1 | PTS system galactitol-specific EIIC component |
| 2812328 | 5934 | IslandPick |  | MACIJPLA_02702 | 2810874 | 2811158 | 1 | hypothetical protein |
| 2812328 | 5934 | IslandPick | gatA_2 | MACIJPLA_02703 | 2811169 | 2811627 | 1 | PTS system galactitol-specific EIIA component |
| 2812328 | 5934 | IslandPick | sorC_2 | MACIJPLA_02704 | 2811679 | 2812644 | -1 | Sorbitol operon regulator |
| 3218692 | 12276 | IslandPick |  | MACIJPLA_03058 | 3206846 | 3207364 | -1 | hypothetical protein |
| 3218692 | 12276 | IslandPick |  | MACIJPLA_03059 | 3207424 | 3215337 | -1 | hypothetical protein |
| 3218692 | 12276 | IslandPick |  | MACIJPLA_03060 | 3215362 | 3215982 | -1 | hypothetical protein |
| 3218692 | 12276 | IslandPick | xerD_3 | MACIJPLA_03061 | 3216301 | 3216930 | -1 | Tyrosine recombinase XerD |
| 3218692 | 12276 | IslandPick | xerD_4 | MACIJPLA_03062 | 3217679 | 3218248 | 1 | Tyrosine recombinase XerD |
| 3218692 | 12276 | IslandPick | dsdC | MACIJPLA_03063 | 3218644 | 3219591 | -1 | HTH-type transcriptional regulator DsdC |
| 3649925 | 13454 | IslandPick |  | MACIJPLA_03458 | 3637210 | 3637710 | 1 | hypothetical protein |
| 3649925 | 13454 | IslandPick |  | MACIJPLA_03459 | 3637769 | 3639307 | 1 | hypothetical protein |
| 3649925 | 13454 | IslandPick |  | MACIJPLA_03460 | 3639327 | 3640664 | 1 | hypothetical protein |
| 3649925 | 13454 | IslandPick |  | MACIJPLA_03461 | 3640661 | 3641326 | 1 | hypothetical protein |
| 3649925 | 13454 | IslandPick | ompA_2 | MACIJPLA_03462 | 3641339 | 3642991 | 1 | Outer membrane protein A |
| 3649925 | 13454 | IslandPick | hcpA_3 | MACIJPLA_03463 | 3643049 | 3643540 | 1 | Major exported protein |
| 3649925 | 13454 | IslandPick | clpV1_2 | MACIJPLA_03464 | 3643731 | 3646367 | 1 | Protein ClpV1 |
| 3649925 | 13454 | IslandPick |  | MACIJPLA_03465 | 3646379 | 3648688 | 1 | hypothetical protein |
| 3649925 | 13454 | IslandPick |  | MACIJPLA_03466 | 3648700 | 3651063 | 1 | hypothetical protein |
| 3675675 | 25733 | IslandPick |  | MACIJPLA_03466 | 3648700 | 3651063 | 1 | hypothetical protein |
| 3675675 | 25733 | IslandPick |  | MACIJPLA_03467 | 3651060 | 3651917 | 1 | hypothetical protein |
| 3675675 | 25733 | IslandPick |  | MACIJPLA_03468 | 3652381 | 3654894 | 1 | hypothetical protein |
| 3675675 | 25733 | IslandPick |  | MACIJPLA_03469 | 3654916 | 3655518 | 1 | hypothetical protein |
| 3675675 | 25733 | IslandPick |  | MACIJPLA_03470 | 3655520 | 3656071 | 1 | hypothetical protein |
| 3675675 | 25733 | IslandPick |  | MACIJPLA_03471 | 3656149 | 3657363 | 1 | hypothetical protein |
| 3675675 | 25733 | IslandPick |  | MACIJPLA_03472 | 3657363 | 3660755 | 1 | hypothetical protein |
| 3675675 | 25733 | IslandPick |  | MACIJPLA_03473 | 3660721 | 3662358 | 1 | hypothetical protein |
| 3675675 | 25733 | IslandPick |  | MACIJPLA_03474 | 3662364 | 3663782 | 1 | hypothetical protein |
| 3675675 | 25733 | IslandPick |  | MACIJPLA_03475 | 3663775 | 3664197 | 1 | hypothetical protein |
| 3675675 | 25733 | IslandPick |  | MACIJPLA_03476 | 3664757 | 3666517 | 1 | hypothetical protein |
| 3675675 | 25733 | IslandPick |  | MACIJPLA_03477 | 3666481 | 3667560 | 1 | hypothetical protein |
| 3675675 | 25733 | IslandPick |  | MACIJPLA_03478 | 3667541 | 3668077 | 1 | hypothetical protein |
| 3675675 | 25733 | IslandPick |  | MACIJPLA_03479 | 3668081 | 3668509 | 1 | hypothetical protein |
| 3675675 | 25733 | IslandPick |  | MACIJPLA_03480 | 3668509 | 3669885 | 1 | hypothetical protein |
| 3675675 | 25733 | IslandPick |  | MACIJPLA_03481 | 3670185 | 3671132 | -1 | Hydroxypyruvate reductase |
| 3675675 | 25733 | IslandPick | kdsD_1 | MACIJPLA_03482 | 3671204 | 3671800 | -1 | Arabinose 5-phosphate isomerase KdsD |
| 3675675 | 25733 | IslandPick | malY_2 | MACIJPLA_03483 | 3671803 | 3672978 | -1 | Protein MalY |
| 3675675 | 25733 | IslandPick | malX_2 | MACIJPLA_03484 | 3672978 | 3674558 | -1 | PTS system maltose-specific EIICB component |
| 3675675 | 25733 | IslandPick | licT | MACIJPLA_03485 | 3674590 | 3675414 | -1 | Transcription antiterminator LicT |
| 3675675 | 25733 | IslandPick | amiC | MACIJPLA_03486 | 3675671 | 3676924 | -1 | N-acetylmuramoyl-L-alanine amidase AmiC |
| 3844474 | 16573 | IslandPick |  | MACIJPLA_03623 | 3827958 | 3828248 | -1 | hypothetical protein |
| 3844474 | 16573 | IslandPick | kpsF | MACIJPLA_03624 | 3829044 | 3830027 | 1 | Arabinose 5-phosphate isomerase KpsF |
| 3844474 | 16573 | IslandPick |  | MACIJPLA_03625 | 3830099 | 3831247 | 1 | hypothetical protein |
| 3844474 | 16573 | IslandPick | kpsD | MACIJPLA_03626 | 3831271 | 3832947 | 1 | Polysialic acid transport protein KpsD |
| 3844474 | 16573 | IslandPick | kpsU | MACIJPLA_03627 | 3832957 | 3833697 | 1 | 3-deoxy-manno-octulosonate cytidylyltransferase |
| 3844474 | 16573 | IslandPick |  | MACIJPLA_03628 | 3833694 | 3835721 | 1 | hypothetical protein |
| 3844474 | 16573 | IslandPick |  | MACIJPLA_03629 | 3835756 | 3836994 | 1 | hypothetical protein |
| 3844474 | 16573 | IslandPick |  | MACIJPLA_03630 | 3837043 | 3838032 | -1 | hypothetical protein |
| 3844474 | 16573 | IslandPick |  | MACIJPLA_03631 | 3838151 | 3841660 | -1 | hypothetical protein |
| 3844474 | 16573 | IslandPick | kpsT | MACIJPLA_03632 | 3841847 | 3842524 | -1 | Polysialic acid transport ATP-binding protein KpsT |
| 3844474 | 16573 | IslandPick | kpsM | MACIJPLA_03633 | 3842521 | 3843297 | -1 | Polysialic acid transport protein KpsM |
| 3844474 | 16573 | IslandPick | epsM | MACIJPLA_03634 | 3844469 | 3845005 | -1 | Type II secretion system protein M |
| 3853976 | 8004 | IslandPick |  | MACIJPLA_03635 | 3845007 | 3846185 | -1 | hypothetical protein |
| 3853976 | 8004 | IslandPick | gspK_1 | MACIJPLA_03636 | 3846182 | 3847159 | -1 | Putative type II secretion system protein K |
| 3853976 | 8004 | IslandPick | xcpW | MACIJPLA_03637 | 3847156 | 3847761 | -1 | Type II secretion system protein J |
| 3853976 | 8004 | IslandPick |  | MACIJPLA_03638 | 3847758 | 3848129 | -1 | hypothetical protein |
| 3853976 | 8004 | IslandPick | epsH | MACIJPLA_03639 | 3848126 | 3848689 | -1 | Type II secretion system protein H |
| 3853976 | 8004 | IslandPick | epsG | MACIJPLA_03640 | 3848693 | 3849148 | -1 | Type II secretion system protein G |
| 3853976 | 8004 | IslandPick | epsF_2 | MACIJPLA_03641 | 3849165 | 3850388 | -1 | Type II secretion system protein F |
| 3853976 | 8004 | IslandPick | epsE_2 | MACIJPLA_03642 | 3850388 | 3851881 | -1 | Type II secretion system protein E |
| 3853976 | 8004 | IslandPick | gspD2 | MACIJPLA_03643 | 3851881 | 3853941 | -1 | Secretin GspD 2 |
| 3853976 | 8004 | IslandPick | gspC2 | MACIJPLA_03644 | 3853971 | 3854930 | -1 | Type II secretion system protein C 2 |
| 4416794 | 5392 | IslandPick | hemR | MACIJPLA_04196 | 4409673 | 4411655 | -1 | Hemin receptor |
| 4416794 | 5392 | IslandPick | hmuT | MACIJPLA_04197 | 4412339 | 4413253 | 1 | Hemin-binding periplasmic protein HmuT |
| 4416794 | 5392 | IslandPick | chuW | MACIJPLA_04198 | 4413273 | 4414610 | 1 | Anaerobilin synthase |
| 4416794 | 5392 | IslandPick | hutX | MACIJPLA_04199 | 4414623 | 4415117 | 1 | Intracellular heme transport protein HutX |
| 4416794 | 5392 | IslandPick |  | MACIJPLA_04200 | 4415117 | 4415740 | 1 | hypothetical protein |
| 4416794 | 5392 | IslandPick | hmuU | MACIJPLA_04201 | 4415825 | 4416781 | 1 | Hemin transport system permease protein HmuU |
| 4416794 | 5392 | IslandPick | hmuV | MACIJPLA_04202 | 4416778 | 4417548 | 1 | Hemin import ATP-binding protein HmuV |
| 4541853 | 5198 | IslandPick | sadB | MACIJPLA_04307 | 4536945 | 4537625 | 1 | Inner membrane lipoprotein SadB |
| 4541853 | 5198 | IslandPick |  | MACIJPLA_04308 | 4537669 | 4541793 | 1 | hypothetical protein |
| 4602480 | 6720 | IslandPick |  | MACIJPLA_04362 | 4595802 | 4596482 | -1 | hypothetical protein |
| 4602480 | 6720 | IslandPick | mak_2 | MACIJPLA_04363 | 4596463 | 4597395 | -1 | Fructokinase |
| 4602480 | 6720 | IslandPick | fbaA_2 | MACIJPLA_04364 | 4597443 | 4598303 | -1 | putative fructose-bisphosphate aldolase |
| 4602480 | 6720 | IslandPick | kbaY_3 | MACIJPLA_04365 | 4598384 | 4599235 | -1 | D-tagatose-1,6-bisphosphate aldolase subunit KbaY |
| 4602480 | 6720 | IslandPick | fruA_2 | MACIJPLA_04366 | 4599247 | 4600338 | -1 | PTS system fructose-specific EIIB'BC component |
| 4602480 | 6720 | IslandPick | frwD_2 | MACIJPLA_04367 | 4600363 | 4600677 | -1 | PTS system fructose-like EIIB component 3 |
| 4602480 | 6720 | IslandPick | fruA_3 | MACIJPLA_04368 | 4600695 | 4601165 | -1 | PTS system fructose-specific EIIABC component |
| 4602480 | 6720 | IslandPick | manR | MACIJPLA_04369 | 4601192 | 4602745 | -1 | Transcriptional regulator ManR |
| 4798207 | 4786 | IslandPick | codB_2 | MACIJPLA_04543 | 4792986 | 4794209 | -1 | Cytosine permease |
| 4798207 | 4786 | IslandPick | atzF | MACIJPLA_04544 | 4794235 | 4794624 | -1 | Allophanate hydrolase |
| 4798207 | 4786 | IslandPick | arcC1_4 | MACIJPLA_04545 | 4794641 | 4795597 | -1 | Carbamate kinase 1 |
| 4798207 | 4786 | IslandPick |  | MACIJPLA_04546 | 4795590 | 4797065 | -1 | hypothetical protein |
| 4798207 | 4786 | IslandPick | fdrA_3 | MACIJPLA_04547 | 4797011 | 4798570 | -1 | Protein FdrA |
